# Panax notoginseng saponins reverse P-gp-mediated steroid resistance in lupus: involvement in the suppression of the SIRT1/FoxO1/MDR1 signalling pathway in lymphocytes

**DOI:** 10.1186/s12906-021-03499-5

**Published:** 2022-01-12

**Authors:** Feng Pan, Yue-jin Li, Ying Lu

**Affiliations:** 1 Department of Immunology and Inflammation, The Second Clinical Medical College of Zhejiang Chinese Medical University, Hangzhou, 310014 Zhejiang China; 2 Department of Immunology and Inflammation, Shanxi Hospital of Integrated Traditional and Western Medicine, Taiyuan, 030000 Shanxi China; 3 Department of Immunology and Inflammation, Zhejiang Academy of Traditional Chinese Medicine, Hangzhou, 310007 Zhejiang China

**Keywords:** Panax notoginseng saponins, P-glycoprotein, Steroid resistance, Lupus nephritis, SIRT1/FoxO1/MDR1 signalling pathway, Th17 cells

## Abstract

**Background:**

P-glycoprotein (P-gp)-mediated steroid resistance (SR) has been suggested to play a significant role in lupus nephritis (LN) treatment failure. Panax notoginseng saponins (PNS), the main effective components of the traditional Chinese medicine notoginseng, exhibited potent reversal capability of P-gp-mediated SR, but its mechanism remains unknown. This study aimed to investigate the effect of PNS on reversing SR in lupus and its underlying mechanism in vivo and in vitro.

**Methods:**

In this study, an SR animal and splenic lymphocyte model were established using low-dose methylprednisolone (MP). Flow cytometry was used to detect the effect of PNS on reversing P-gp-mediated SR and the expression of P-gp in different T-cells phenotypes. Serum levels of ANA and dsDNA in lupus mice were measured by ELISA. Apoptosis was identified by Annexin V-FITC/PI staining. RT–PCR and Western blotting were used to detect the protein and mRNA expression levels of SIRT1, FoxO1, and MDR1 in SR splenic lymphocytes from lupus mice (SLCs/MPs).

**Results:**

PNS could reverse the SR in lupus mice. Simultaneously, PNS increased the apoptotic effect of MP on SLCs/MP cells. The increased accumulation of rhodamine-123 (Rh-123) indicated that intracellular steroid accumulation could be increased by the action of PNS. Moreover, PNS decreased the expression of P-gp levels. Further experiments elucidated that the SIRT1/FoxO1/MDR1 signalling pathway existed in SLCs/MP cells, and PNS suppressed its expression level to reverse SR. The expression of P-gp in Th17 from SLCs/MP cells was increased, while PNS could reduce its level in a more obvious trend.

**Conclusion:**

The present study suggested that PNS reversed P-gp-mediated SR via the SIRT1/FoxO1/MDR1 signalling pathway, which might become a valuable drug for the treatment of SR in lupus. Th17 might be the main effector cell of PNS reversing SR.

**Supplementary Information:**

The online version contains supplementary material available at 10.1186/s12906-021-03499-5.

## Background

Systemic lupus erythematosus (SLE) is a highly heterogeneous systemic autoimmune disease characterized by the interaction between autoimmune T lymphocytes and overactivated B lymphocytes, producing a large number of autoantibodies and immune complexes, which has complicated clinical presentations and involves multiple systems and organs [1, 2]. Lupus nephritis(LN) is the most common and clinically challenging complication in SLE. Steroids, a basic drug for the treatment of lupus, can directly act on lymphocytes, inhibit antibody formation, effectively prevent inflammatory reactions, quickly relieve clinical symptoms, and prevent the further progression of disease [3, 4]. Nevertheless, steroid resistance (SR) is often observed in the treatment of lupus, leading to poor therapeutic effects and susceptibility to recurrence [5, 6].

The SR mechanisms involve many factors, including P-glycoprotein (P-gp), glucocorticoid receptor(GR) polymorphisms, macrophage migration inhibitory factor (MIF), and Th17 cells [7, 8]. Recently, GR polymorphisms such as NR3C1(rs41423247, rs6189/rs6190 and rs56149945) have been identified to determine steroid sensitivity in several autoimmune diseases through reducing the GR complex and affecting the pharmacological effects of steroid [9]. MIF is an inflammatory cytokine that actively participates in multiple stages of the inflammatory response, which can impair steroid sensitivity by counter-regulating steroid induced expression of MAPK phosphatase-1 (MKP-1) that suppresses the secretion of pro-inflammatory cytokine [10]. SR is closely related to Thl7 cells, and one study found that human Thl7 cells responded poorly to steroid. Studies have shown that one of the key mechanisms of immunosuppressants such as cyclosporine A and tacrolimus on immune response and SR is the selective inhibition of Th17 cells [11, 12].

Among these factors, P-gp, an ATP-binding cassette member produced by the multidrug resistance 1 (*MDR1*) gene, is the chief potential mechanism [13–15]. Acting as an efflux pump, P-gp effectively transports steroids from intracellular fluid to the extracellular environment through membranes, reducing intracellular steroid concentrations and leading to SR [16–18]. Meanwhile, the expression of P-gp in peripheral blood lymphocytes of lupus patients has individual differences and is significantly higher in patients with good steroid effects than in patients with poor curative effects. Moreover, the expression level of P-gp is positively correlated with disease activity [7, 19]. For these reasons, it is necessary to find an effective P-gp inhibitor, which might be a critical solution to reverse SR in lupus.

Forkhead box-containing protein of the O subfamily 1 (FoxO1), a forkhead box class-O transcription factor, can affect the level of P-gp by adjusting the transcription of the *MDR1* gene [20]. The study showed that when FoxO1 was deacetylated, it shuttled into the nucleus of the cell and initiated the transcription of *MDR1*, resulting in an increase in P-gp expression. Silent information regulation factor related enzyme 1 (SIRT1) is a nicotinamide adenine dinucleotide (NAD)–dependent histone dehydrogenase associated with histone and nonhistone acetylation [21]. More significantly, FoxO1 is the substrate for deacetylation of SIRT1, which promotes nuclear accumulation of FoxO1 and thereby prolongs the transcription of downstream related genes in the nucleus, such as *MDR1* [22, 23]. The SIRT1/FoxO1/MDR1 signalling pathway has been confirmed in drug resistance of tumour cells [20, 24], but there is little research on the field of SR in lupus. Accordingly, we speculate that this signalling pathway might exist and play significant roles in the molecular mechanisms of SR in lupus.

Natural products have long been and are still the source of treatments for different serious disorders [25–28]. Panax notoginseng saponin (PNS) is the main bioactive compound isolated from the traditional Chinese medicine Panax notoginseng. PNS has a wild range of pharmacological activities, such as anti-inflammatory and anticancer activities, expanding blood vessels, reducing oxygen consumption in the myocardium, inhibiting platelet aggregation, and regulating blood glucose [29–32]. In addition, PNS and its composition have an obvious ability to reverse multiple drug resistance. Similar results have been reported in many studies using animals and cells [33–35]. PNS could inhibit P-glycoprotein-mediated multidrug resistance in tumour cells [36]. In nude mice bearing A549/T tumours, PNS and docetaxel treatment significantly suppressed the growth of drug-resistant tumours without an increase in toxicity when compared to docetaxel given alone at the same dose [37]. Moreover, PNS attenuated ultraviolet B-induced glucocorticoid insensitivity through the Nrf2/HDAC2 pathway [38].

In our previous study, we proved that PNS can reduce the human lymphocyte P-gp expression level and inhibit its activity, which is more effective than the classic P-gp inhibitor verapamil [39]. Although PNS has great potential to reverse drug resistance, the mechanism of reversing SR in lupus has not been reported. In the present study, we aimed to investigate the effect of PNS on reversing SR in lupus mice and determine whether PNS reverses P-gp-mediated SR via SIRT1/FoxO1/MDR1 signalling in lymphocytes of lupus mice, which may be the major mechanism underlying the SR reversal potential of PNS in lupus.

## Methods

### Chemicals and reagents

Mouse red blood cell lysis buffer was purchased from Tianjin Haoyang Biotech Co., Ltd. (Tianjin, China). Methylprednisolone (MP) for injection was obtained from Pfizer (New York, USA). Notoginseng total saponin was provided by Guangxi Wuzhou Pharmaceutical (group) Co., Ltd. (Guangxi, China). Tariquidar (TQR) was provided by Selleckchem (Houston, TX, USA). Serum ANA and dsDNA ELISA kits were purchased from Cusabio Biotech Co., Ltd. (Wuhan, China). RPMI-1640 was obtained from Ginuo Biological Pharmaceutical Technology Co. Ltd. (Hangzhou, China). Foetal bovine serum was obtained from Hangzhou Sijiqing Biological Engineering Materials Co., Ltd. (Hangzhou, China). Rhodamine 123 (Rh-123) and concanavalin A (ConA) were acquired from Sigma–Aldrich (St. Louis, MO, USA). Primary antibodies against P-gp, CD69, SIRT1, FoxO1, CD4, CD8, CD25, IL-17A, IFN-γ, IL-4 and GAPDH were purchased from Abcam (Cambridge, MA, USA). The secondary antibody Annexin V-FITC apoptosis detection kit was purchased from Beijing Jiamei Biotech Co., Ltd. (Beijing, China). SIRT1 siRNA and PCR primers were developed and synthesized by GenePharma (Shanghai, China).

### Animals

Female NZW/LacJ mice (aged 16 weeks, weighing 25–30 g) were imported from Jackson Laboratory by the Model Animal Research Center of Nanjing University (Nanjing, China). The mice were constituted under specific pathogen-free (SPF) conditions. All experiments were approved by the Animal Ethics Committee of Zhejiang Academy of Traditional Chinese Medicine (Zhejiang, China).

### Establishment and grouping of the animal model

First, the SR lupus mouse (SRLM) model was established by intraperitoneal injection of low-dose MP for four weeks (0.8 mg·kg^−1^·day^−1^, *n* = 30). Compared with untreated mice, the criterion for the successful construction of the SRLM model was that the expression of P-gp increased and the accumulation of Rh-123 decreased in the splenic lymphocytes of lupus mice (SLCs). Then, the SRLM were randomly divided into five group: SR control group (0.8 mg·kg^−1^·day^−1^ MP, 4 weeks, *n* = 6), SR with high-dose MP group (0.8 mg·kg^−1^·day^−1^ MP, 12 mg·kg^−1^·day^−1^ MP, 4 weeks, *n* = 6), SR with high-dose MP and high-dose PNS group (0.8 mg·kg^−1^·day^−1^ MP, 12 mg·kg^−1^·day^−1^ MP, 100 mg·kg^−1^·day^−1^,4 weeks, *n* = 6), SR with high-dose MP and low-dose PNS group (0.8 mg·kg^−1^·day^−1^ MP, 12 mg·kg^−1^·day^−1^ MP, 50 mg·kg^−1^·day^−1^, 4 weeks, *n* = 6) and SR with high-dose MP and P-gp inhibitor (TQR) group (0.8 mg·kg^−1^·day^−1^ MP, 8 mg·kg^−1^·day^−1^ TQR, 4 weeks, *n* = 6). At the end of the research, the mice were euthanized through CO_2_ asphyxiation using slow displacement of chamber air with compressed CO_2_ (25%/min), and their serum and spleen were harvested. All spleen tissues were immediately used to prepare SLCs. The expression of P-gp and the accumulation of Rh-123 in SLCs from each group were measured by flow cytometry.

### Preparation of SLCs

After euthanization, the mice were soaked in 75% ethanol for 20 s. Spleens were removed from the abdominal cavity at a superclean worktable, placed into 300 nylon meshes and immersed in a culture dish containing 10 mL RPMI-1640 medium. Spleen tissue was gently ground with the syringe plunger until white connective tissue was visible to the naked eye. The suspension was transferred to new centrifuge tubes and centrifuged at 1200 rpm for 5 min at 4 °C. After pouring the supernatant, 2 ml red blood cell lysis buffer was added into centrifuge tubes and placed on ice for 5 min. The suspension was then centrifuged at 1500 rpm for 5 min at 4 °C. The layer in the bottom of the centrifuge tube was collected and resuspended in RPMI-1640 medium containing 100 U/ml penicillin, 100 mg/ml streptomycin, and 10% FBS. The suspension was then cultured at 37 °C in a humidified atmosphere of 5% CO_2_. Trypan blue staining was used to estimate the viability of SLCs.

### Establishment of steroid-resistant SLCs (SLCs/MPs) and cell culture

According to our previous study [40], we utilized a small dose of MP (2 μg/ml) to induce SLCs for 72 h. The successful standard of constructing SLCs/MP cells was that the expression of P-gp was upregulated and the accumulation of rhodamine-123 in cells was downregulated compared with untreated SLCs. Then, the SLCs/MP cells were treated with RPMI-1640 alone or in combination with PNS (100, 200 μg/ml) or with SIRT1-siRNA transfection. The cells were cultured at 37 °C in a humidified atmosphere of 5% CO_2_ for 72 h.

### Transfection of siRNA and lentivirus targeted to SIRT1

SLCs/MP cells (5.0 × 10^4^/ml) were transfected with specific siRNAs and lentivirus through use of oligo-siRNA-Mate and polybrene compound (siRNA sequence, transfection reagent and lentivirus were obtained from Genepharma Company) according to the manufacturer’s guidelines. Sequences of siRNAs were as follows: si-SIRT1 5’- GAUGAAGUUGACCUCCUCATT-3’; Negative control siRNA 5’- UUCUCCGAACGUGUCACGUTT -3’. After transfection, cells were cultivated at 37 °C in RPMI-1640 with 10% FBS for 72 h. Then, the cells were collected, and the intervention efficiency was determined by RT–PCR.

### Flow cytometry

To detect the reversal capability of PNS on SLCs/MP cells, an Annexin V-FITC/PI apoptosis detection kit (Beijing Jiamei Biotech, Beijing, China) was used to investigate cell apoptosis induced by MP. After preincubating the SLCs/MP cells with ConA alone, ConA containing PNS (100 μg/ml, 200 μg/ml), or si-SIRT for 24 h, the cells were cultured with MP (200 μg/ml) for an additional 72 h. After incubation, cells were collected and washed with ice-cold PBS three times and resuspended at a concentration of 3 × 10^5^ cells/mL in 200 μL of binding buffer. The samples were then subsequently treated with 5 μL of Annexin V-FITC/PI at room temperature in the dark for 15 min and detected within 1 h by flow cytometry (FACS FC500; Beckman Coulter, Brea, CA, USA).

For the CD69 expression assay, cells were cultivated with anti-CD69 antibody (0.1 μg/ml) at room temperature for 30 min and FITC-conjugated goat anti-mouse monoclonal antibody (1:20 diluted) at room temperature for 30 min successively. Flow cytometry was used to detect CD69 expression.

The potential of PNS to reverse P-gp expression was determined in the SLCs after animal experiment intervention and SLCs/MP cells. Cells (3.0 × 10^5^/ml) were cultivated with anti-P-gp antibody (0.1 μg/ml) at room temperature for 30 min. After washing twice with ice-cold PBS, cells were cocultured with FITC-conjugated goat anti-mouse monoclonal antibody (1:20 diluted) at room temperature for 30 min. Then, ice-cold PBS was used to wash the cells two times, and the level of fluorescent staining was measured by flow cytometry. Isotype-matched FITC-conjugated irrelevant antibodies were used as negative controls.

As the substrate of P-gp, the cumulative detection of Rh-123 was used to reflect the reversal effect of PNS on P-gp-mediated RS. Cells (3.0 × 10^5^/ml) were cultivated with 5 μg/ml Rh-123 for 30 min at 37 °C and collected by washing twice with cold PBS and centrifugation. The intracellular mean fluorescence was measured using a flow cytometer with an excitation wavelength of 488 nm and emission wavelength of 530 nm.

To determine the main effector cells of PNS reversing SR, the expression of P-gp in CD8 + T cells, Th1, Th2, Th17 and Treg cells from SLCs and SLCs/MP was detected by flow cytometry. FITC anti-mouse CD4, PE anti-mouse CD8 and PC5 anti-mouse CD25 were used for cell surface staining. ECD anti-mouse IL-17A, APC700 anti-mouse IFN-γ and PB anti-mouse IL-4 were used for intracellular staining after fixation and permeabilization.

FlowJo vX0.7 software (Tree Star, Inc., San Carlos, CA) was used to analyse flow cytometry data.

### Enzyme-linked immunosorbent assay

Serum samples were centrifuged from whole blood at 3500 × g for 10 min at 4 °C after a 30-min undisturbed separation at room temperature. The concentrations of ANA and anti-dsDNA antibodies in serum were detected by using an enzyme-linked immunosorbent assay (ELISA) kit according to the manufacturer’s instructions.

### Quantitative real-time polymerase chain reaction analysis

Total RNA of each group was isolated using a GenePharma total RNA kit (GenePharma, Shanghai, China) in accordance with the operational guidelines. The concentration of the total RNA was assessed by a NanoDrop spectrophotometer (NanoDrop 2000, MA, USA). cDNA synthesis was performed with a PrimeScript™ RT reagent kit (TaKaRa, Dalian, China) under the guidance of an operational protocol. Then, qRT–PCR was implemented on a 7500 StepOnePlus™ (Applied Biosystems, Foster, CA, USA) using a SYBR® RPremix Ex Taq™ Kit (TaKaRa, Dalian, China). The 2^−△△Ct^ method was used to calculate the relative mRNA expression of target genes (standardized by GAPDH). SIRT1 forward primer: 5’- GGCTACCGAGGTCCATAT.

ACTTTTG-3'; reverse primer: 5'-TCAGGTGGAGGAATTGTTTCTGG-3'). MDR1 forward primer: 5'-GCGTATTTGGGATGTTTCGCTATG-3'; reverse primer: 5'- AC.

CAGCATCAAGAGGGGAAGTAATG-3'). GAPDH forward primer: 5'-GAGAAAC.

CTGCCAAGTATGATGAC-3'; reverse primer: 5'-AGAGTGGGAGTTGCTGTTGA

AG-3').

### Western blot analysis

Pretreated cells were collected, and total cytoplasmic protein and nuclear protein were extracted and quantified using a BCA protein assay kit (Beyotime Biotechnology, Shanghai, China). Analysis of SIRT1 and FoxO1 expression was performed using a Wes simple western instrument (Wes, Protein Simple, Santa Clara, CA). The sample was diluted with 0.1 × Sample Buffer (Protein Simple, CA) and mixed with 5 × fluorescent master mix (Protein Simple, CA) for denaturation. Primary rabbit monoclonal antibodies (Abcam, UK 1:50) were used to detect mouse SIRT1 and FoxO1. GAPDH (Abcam, UK 1:100) was used as an internal standard. Secondary HRP conjugation was provided in the detection module. Then, the immunoassay plate was prepared as prompted by the instructions, and Wes was started. The experiment was repeated three times, and all protein levels were normalized to GAPDH expression.

### Statistical analysis

The analysis process was performed using SPSS 19.0 software (SPSS Inc., Chicago, IL, USA), and measurement data consistent with a normal distribution are expressed as the mean ± standard deviation. Statistical comparisons were performed using one-way analysis of variance (ANOVA) and Student’s t-test. *P* < 0.05 was considered a statistically significant difference. All of the experiments were performed at least three separate times.

## Results

### PNS reverses P-gp-mediated SR in lupus mice

**Fig. 1:**
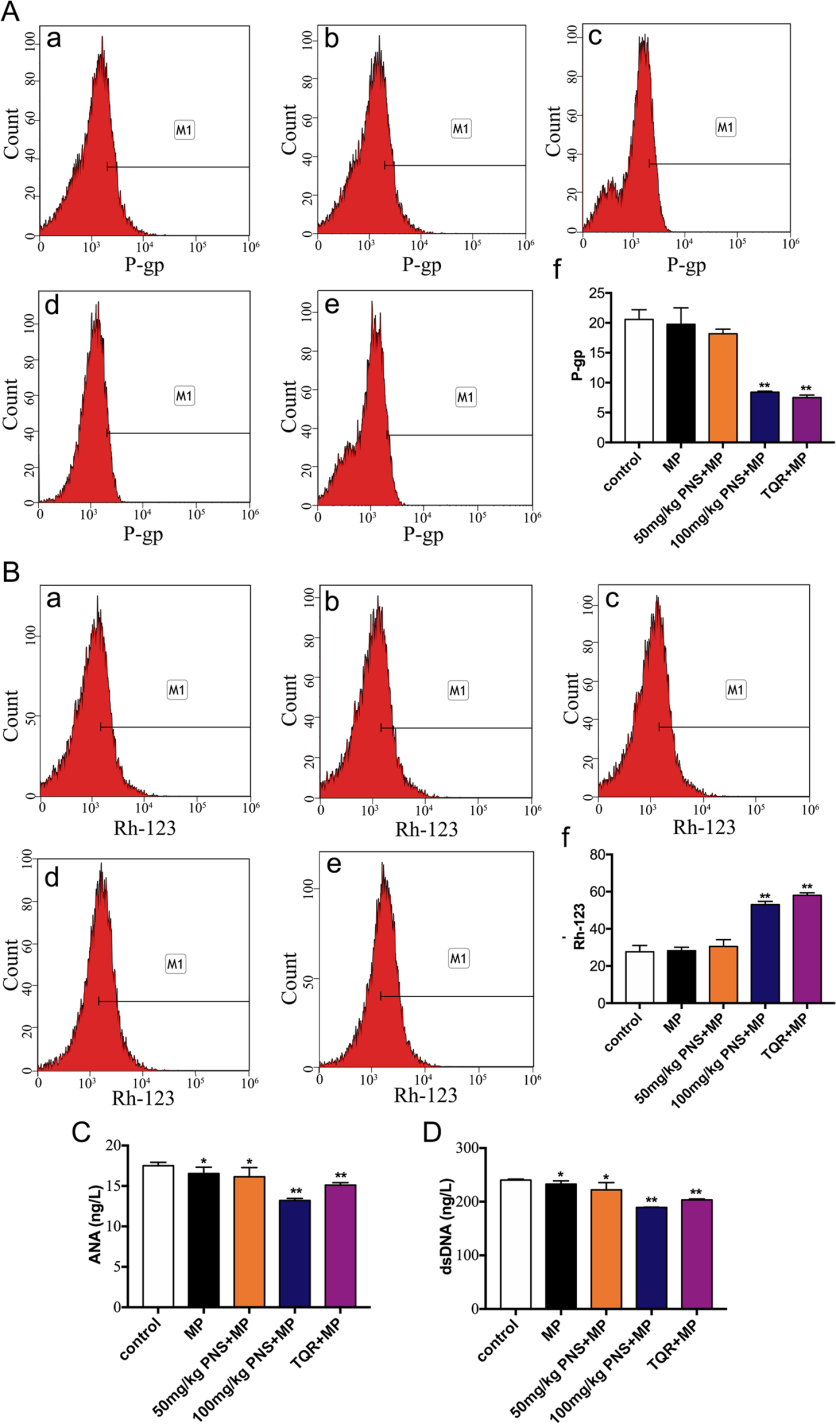
Effect of PNS on reversing P-gp-mediated SR in lupus mice. SRLM treated with low-dose MP is indicated as a control. SRLMs were treated with low-dose MP alone, combination high-dose MP, combination high-dose MP and PNS (50 mg/kg, 100 mg/kg), or combination TQR for 4 weeks. Then, the expression of P-gp and accumulation of Rh-123 in splenic lymphocytes and serum ANA and dsDNA levels were measured by flow cytometry and ELISA. (**A**, **a**)(**B**, **a**) SRLM treated with low-dose MP. (**A**, **b**)(**B**, **b**) SRLM treated with low-dose MP and high-dose MP. (**A**, **c**)(**B**, **c**) SRLM treated with low-dose MP, high-dose MP and PNS (50 mg/kg). (**A**, **d**)(**B**, **d**) SRLM treated with low-dose MP, high-dose MP and PNS (100 mg/kg). (**A**, **e**)(**B**, **e**) SRLM treated with low-dose MP, high-dose MP and TQR. (**A**, **f**) Analysis of P-gp expression. (**B**, **f**) Analysis of intracellular accumulation of Rh-123. (**C**) Analysis of serum ANA level. (**D**) Analysis of serum dsDNA level. Experiments were repeated three times and data are expressed as the means ± SD. *P < 0.05 vs. control. **P < 0.01 vs. control

The SR of lupus was characterized by increased expression of P-gp and decreased accumulation of Rh-123 in lymphocytes, as well as decreased sensitivity to steroids. To determine whether PNS can reverse SR in lupus mice, the expression of P-gp and the accumulation of Rh-123 in splenic lymphocytes from SRLM-treated mice were measured by flow cytometry, and the levels of ANA and dsDNA in serum were examined by ELISA. The flow cytometry results showed that, compared with the SR control group and SR with high-dose MP group, the expression of P-gp was downregulated and the accumulation of rhodamine-123 in splenic lymphocytes was increased in the high-dose PNS and TQR groups; therefore, PNS had a significant ability to reverse SR in lupus mice (Fig. 1A,B). The ELISA results showed that ANA and dsDNA, which reflect the activity of SLE, were decreased to different degrees in the intervention groups treated with high-dose MP or high-dose MP with PNS or TQR. Compared with high-dose MP alone group, combination with PNS group decreased more significantly (Fig. 1C,D). These results indicated that PNS can increase the sensitivity of steroids, and the effect of the combination of PNS and steroids is stronger than that of a single steroid.

### Reversal effects of PNS on sensitivity to MP in SLCs/MP cells

**Fig. 2:**
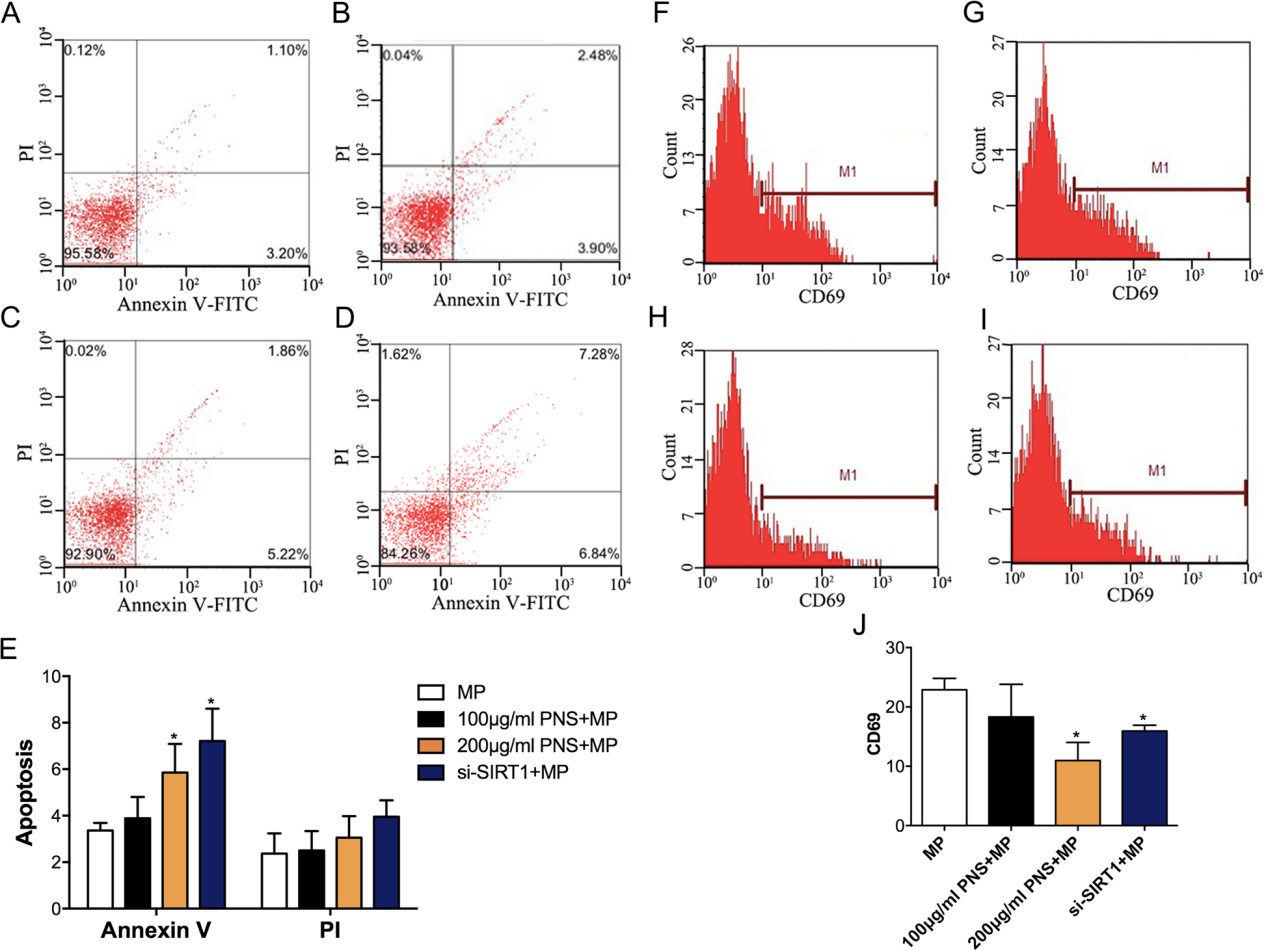
Effect of PNS on MP-induced cell apoptosis and CD69 expression. SLCs/MP cells treated with ConA and MP are indicated as controls. After SLCs/MP cells were treated with ConA alone, ConA containing PNS (100 μg/ml, 200 μg/ml), or si-SIRT for 24 h, the cells were cultured with MP (200 μg/ml) for 72 h, and apoptotic cells were measured. (**A**)(**F**) SLCs/MP cells treated with ConA. (**B**)(**G**) SLCs/MP cells treated with ConA containing 100 μg/ml PNS. (**C**)(**H**) SLCs/MP cells treated with ConA containing 200 μg/ml PNS. (**D**)(**I**) SLCs/MP cells treated with ConA containing si-SIRT1. (**E**)(**J**) Analysis of cell apoptosis and CD69 expression. Experiments were repeated three times and data are expressed as means ± SD. *P < 0.05 vs. control

The influence of PNS on the sensitivity of the MP in the SLCs/MP cells was evaluated through cell apoptosis and CD69 expression assays. Flow cytometry showed that PNS increased the number of apoptotic SLCs/MP cells and decreased the expression of CD69 induced by MP compared with the untreated group (Fig. 2). These findings verified that PNS could enhance the potency of MP against SLCs/MP, supporting the notion that PNS has a reversal effect on the steroid sensitivity of SLCs/MP cells.

### PNS efficiently decreases the expression of P-gp and enhances the accumulation of Rh-123 in SLCs/MP cells

**Fig. 3:**
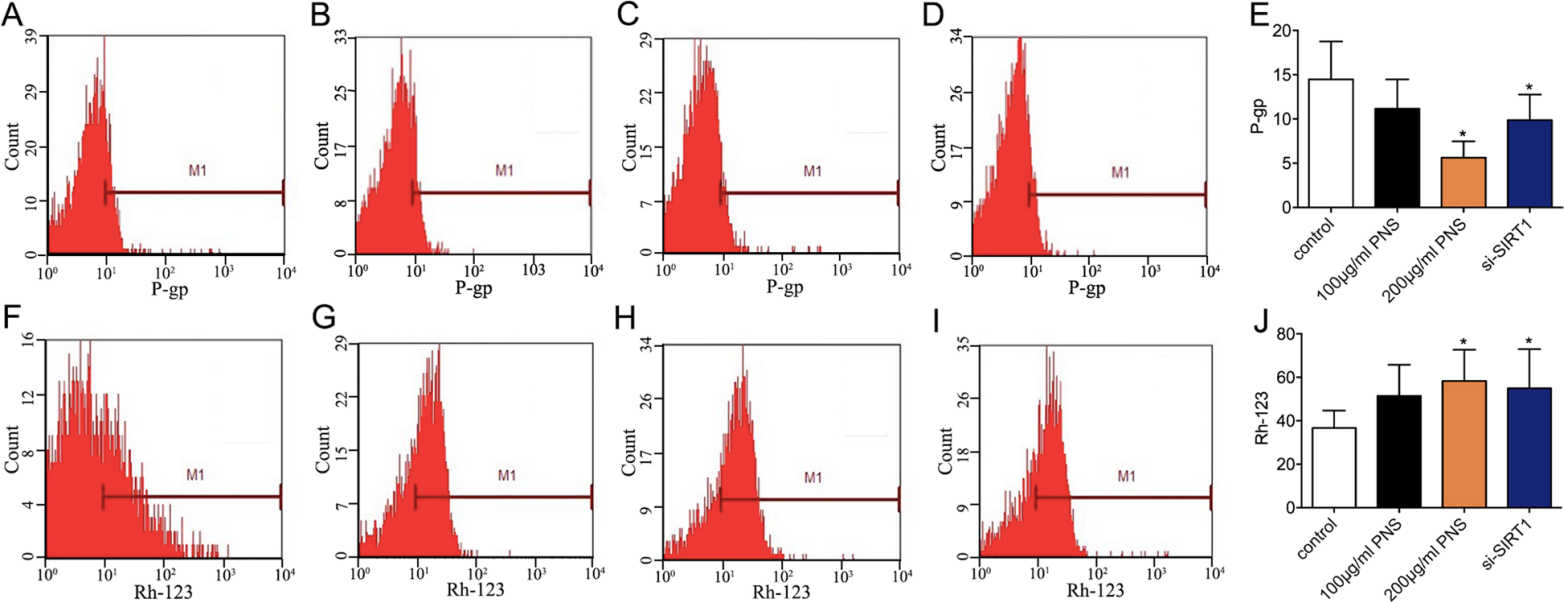
Effect of PNS on the expression of P-gp and the intracellular accumulation of Rh-123 in SLCs/MP cells. SLCs/MP cells cultured with RPMI-1640 alone are indicated as controls. SLCs/MP cells were incubated with RPMI-1640 alone, PNS (100 μg/ml, 200 μg/ml) or si-SIRT1 for 72 h. Then, the P-gp expression and the efflux pump function were determined by flow cytometry. (**A**)(**F**) SLCs/MP cells treated with RPMI-1640 alone. (**B**)(**G**) SLCs/MP cells treated with 100 μg/ml PNS. (**C**)(**H**) SLCs/MP cells treated with 200 μg/ml PNS. (**D**)(**I**) SLCs/MP cells treated with si-SIRT1. (**E**) Analysis of P-gp expression. (**J**) Analysis of intracellular accumulation of Rh-123. Experiments were repeated three times and data are expressed as the means ± SD. *P < 0.05 vs. control

To calculate the P-gp levels, flow cytometry was performed. After incubation with PNS (200 μg/ml) for 72 h, the expression of P-gp in SLCs/MP cells was markedly decreased compared with that in untreated SLCs/MP cells (Fig. 3A-E). The findings indicated that PNS could suppress the expression of P-gp.

The ability of PNS to inhibit P-gp-mediated drug transport was studied using the P-gp substrate rhodamine 123 (Rh-123). Flow cytometry results revealed that PNS enhanced the accumulation of Rh-123 in SLCs/MP cells compared to untreated SLCs/MP cells (Fig. 3F-J). The findings stated that PNS could increase intracellular drug concentrations.

### The SIRT1/FoxO1/MDR1 signalling pathway existed in SLCS/MP cells

**Fig. 4:**
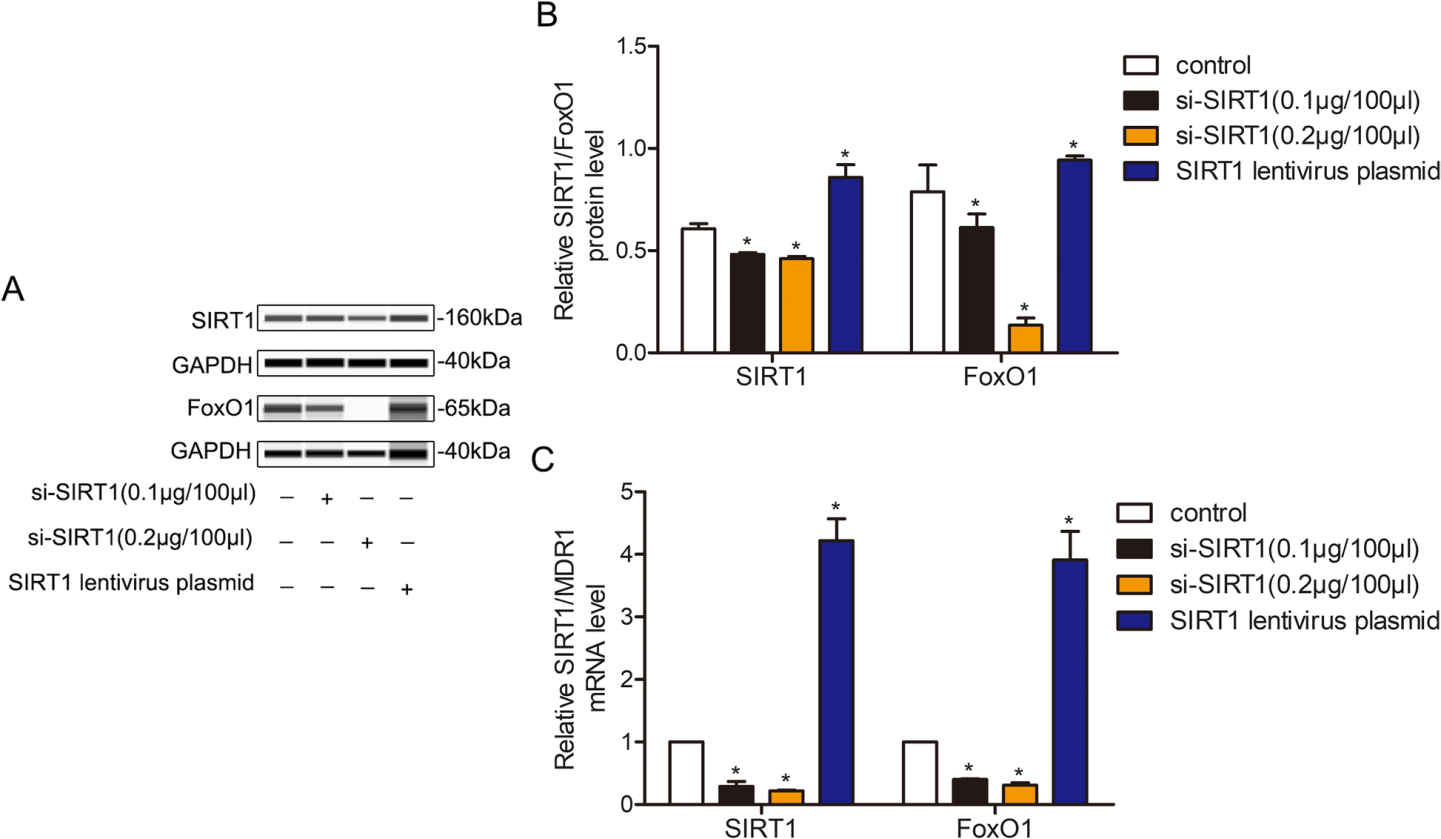
The expression levels of FoxO1 and MDR1 were positively correlated with SIRT1 in SLCs/MP cells. SLCs/MP cells treated with RPMI-1640 were used as controls. SLCs/MP cells were incubated with RPMI-1640, si-SIRT (0.1 μg/100 μl, 0.2 μg/100 μl) or SIRT1 lentivirus plasmid for 72 h. (**A**) Western blot analysis was performed to detect the protein levels of SIRT1 and FoxO1. (**B**) Analysis of protein expression. (**C**) SIRT1 and MDR1 mRNA levels were measured by qRT–PCR. Experiments were repeated three times and data are expressed as the means ± SD. *P < 0.05 vs. control

To investigate the existence of the SIRT1/FoxO1/MDR1 signalling pathway in SLCS/MP cells, SIRT1 was specifically knocked down and overexpressed by gene intervention technology, and the relevant expression of FoxO1 and MDR1 was detected. Western blotting results showed that the levels of cytoplasmic SIRT1 and nuclear FoxO1 in the SIRT1 siRNA group were lower than those in the control group, especially in the high-dose group, and the plasma SIRT1 and nuclear FoxO1 levels in the lentiviral group were higher than those in the control group(Fig. 4A-B, supplementary blots file). qRT–PCR results showed that SIRT1 gene expression was downregulated in the SIRT1 siRNA low- and high-dose groups, especially in the high-dose group, and MDR1 gene expression was also downregulated, while the expression of SIRT1 and MDR1 genes was increased in the lentiviral transfection group (Fig. 4C). These results showed that when the SIRT1 level was changed in SLCs/MP cells, the concentration of FoxO1 in the nucleus, the expression level of the MDR1 gene and the level of RS changed synchronously, which indicated that this signalling pathway might exist in SLCs/MP cells.

### PNS decreases the expression of SIRT1, FoxO1 and MDR1 in SLCs/MP cells

**Fig. 5:**
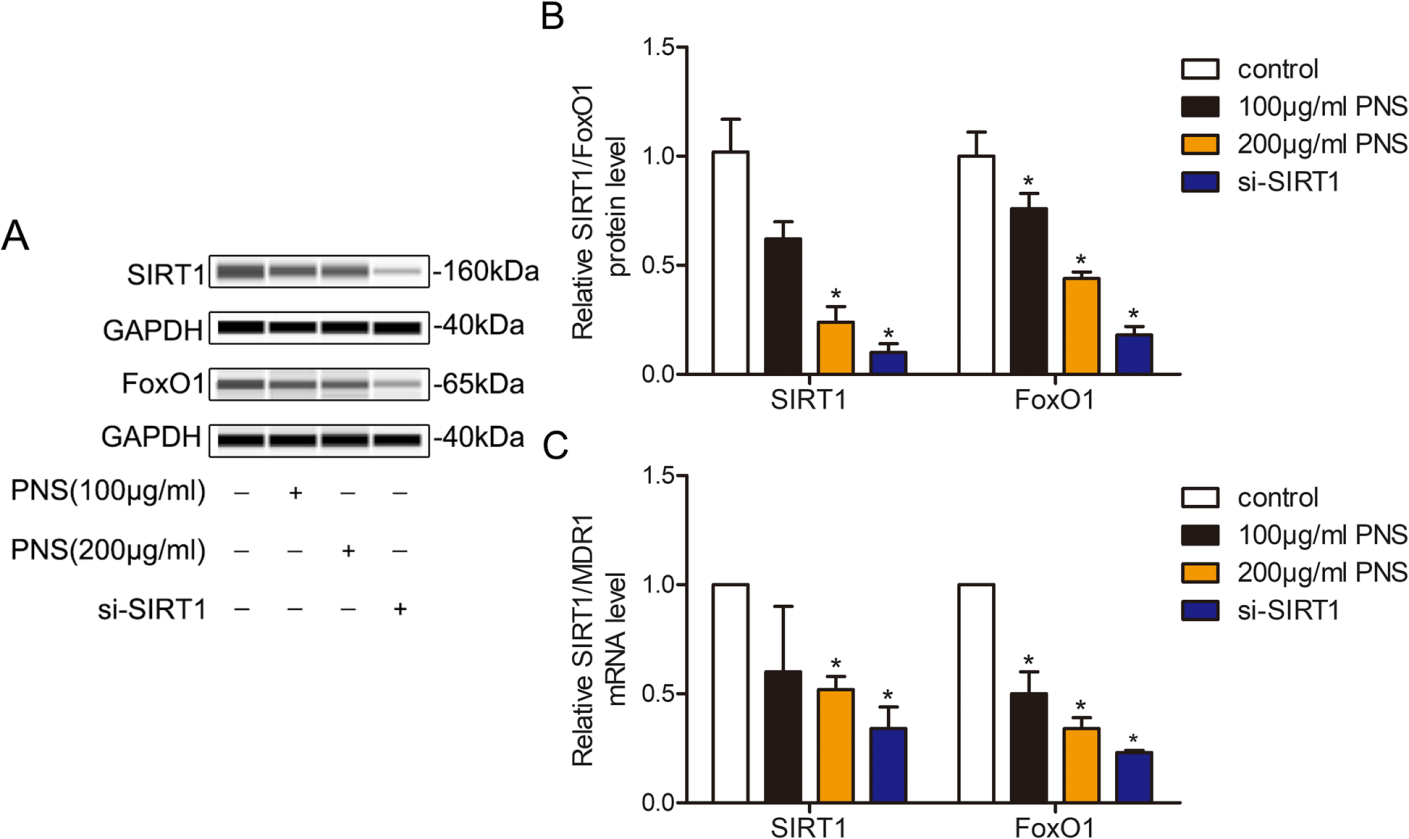
PNS downregulated the expression of P-gp by inhibiting SIRT1/FoxO1/MDR1 signalling pathways in SLCs/MP cells. SLCs/MP cells treated with RPMI-1640 are indicated as controls. The protein levels of SIRT1 and FoxO1 and the mRNA levels of SIRT1 and *MDR1* in SLCs/MP cells incubated with RPMI-1640, PNS (100 μg/ml, 200 μg/ml) or si-SIRT1 for 72 h were assessed using Western blotting and qRT–PCR. (**A**) Western blot analysis was performed to detect the protein levels of SIRT1 and FoxO1. (**B**) Analysis of protein expression. (**C**) qRT–PCR was used to determine SIRT1 and *MDR1* mRNA levels. Experiments were repeated three times and data are expressed as the means ± SD. *P < 0.05 vs. control.

To investigate whether the downregulation of the expression of P-gp by PNS in SLCs/MP cells was associated with the SIRT1/FoxO1/MDR1 pathway, the expression levels of SIRT1, FoxO1 and *MDR1* in SLCs/MP cells were investigated. The Western blotting and qRT–PCR results demonstrated that the mRNA expression levels of SIRT1 and *MDR1* and the protein expression levels of SIRT1 and FoxO1 in SLCs/MP cells were significantly suppressed in comparison with untreated SLCs/MP cells after 72 h of incubation with 200 μg/ml PNS, which is in accordance with SLCs/MP cells transfected with siRNA for SIRT1 (Fig. 5, supplementary blots file). These results indicated that PNS downregulated the expression of P-gp by suppressing the activation of the SIRT1/FoxO1/MDR1 pathway in SLCs/MP cells.

### Th17 is the main effector cell of PNS reversing SR

**Fig. 6:**
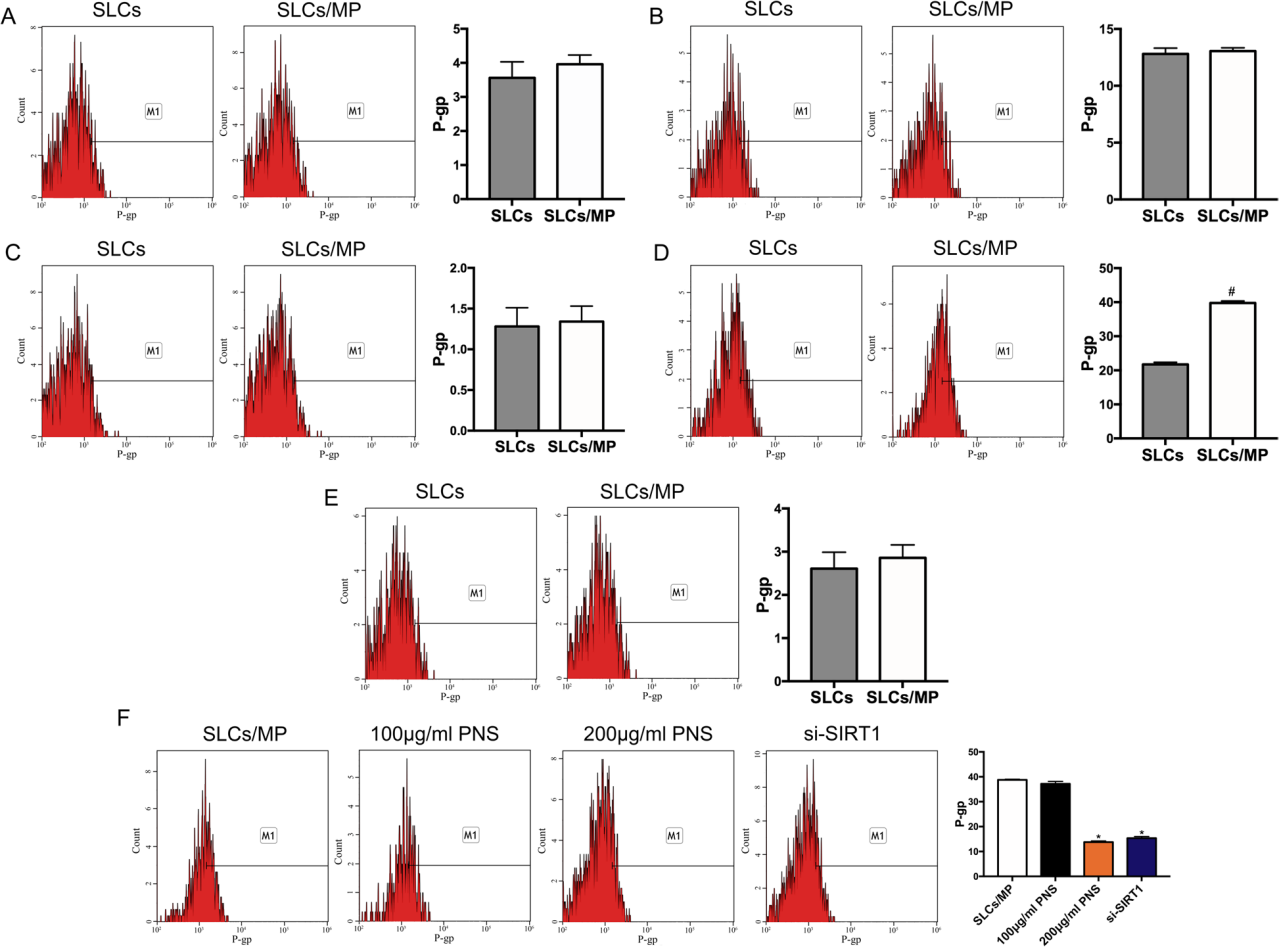
Th17 might be the main effector cell of PNS reversing SR in lupus. The expression of P-gp in different T-cells phenotypes from SLCs and SLCs/MP were detected by flow cytometry. (**A**) The expression of P-gp in CD8 + T cells. (**B**)The expression of P-gp in Th1 cells. (**C**) The expression of P-gp in Th2 cells. (**D**) The expression of P-gp in Th17 cells. (**E**) The expression of P-gp in Treg cells. (**E**) The expression of P-gp in Th17 cells treated by PNS. Experiments were repeated three times and data are expressed as the means ± SD. ^**#**^*P* < 0.05 vs. SLCs. *P < 0.05 vs. SLCs/MP

To determine the main effector cells of PNS reversing SR, the expression of P-gp in different T-cells phenotypes from SLCs and SLCs/MP were analyzed firstly. We found that the level of P-gp was no difference in CD8 + T-cells, Th1, Th2, and Treg cells from SLCs and SLCs/MP cells, while the expression of P-gp was increased in Th17 cells from SLCs/MP cells. Further, PNS significantly decreased the expression of P-gp in Th17 cells from SLCs/MP compared with untreated SLCs/MP cells (Fig. 6, Supplementary figure). These findings indicated that Th17 might be the main effector cell of PNS reversing SR.

## Discussion

SR mediated by drug transporters, especially P-gp, is a significant obstacle to the successful treatment of lupus [41]. The main pathological mechanism of lupus is the proliferation of a large number of lymphocytes, which are also the main intervention target of steroids and immunosuppressive therapy [42]. Thus, directly and effectively blocking the P-gp transport pump or reducing its expression to increase the sensitivity of lymphocytes to steroids may be an optimal strategy for SR reversal in lupus. Currently, the validated approaches for P-gp-mediated SR in lupus include intensive treatments with methylprednisolone (MP) or plasmapheresis [43] and P-gp antagonists [44]. Nevertheless, high-dose steroid shock often overinhibits the immune function of patients and increases the chance of serious infection; furthermore, P-gp antagonists can produce more inevitable side effects. In summary, it is urgent to explore an effective and minimally toxic reversal agent of SR in lupus.

To identify low side effects and efficient P-gp inhibitors, a growing number of researchers have focused their research on the exploration of active ingredients from natural sources that have the ability to restore relevant indicators to normal levels. Many active components of traditional Chinese medicine have been shown to reverse drug resistance by restraining P-gp expression, such as curcumin [45], ginsenoside [46], tetramethylpyrazine [47] and osthole [48]. Our previous studies showed that PNS could inhibit the expression of P-gp without significant cytotoxicity. Taken together, PNS might have great potential to reverse SR and needs to be explored.

In the present study, PNS significantly reduced the expression of P-gp and increased the accumulation of rhodamine-123 (Rh-123) in splenic lymphocytes from SR lupus mice (SRLMs). In addition, PNS can increase the sensitivity to steroids in SRLM, so it can significantly reduce the level of serum ANA and dsDNA expression in SRLM when combined with high-dose steroids. On the basis of animal experiments, we used low-dose methylprednisolone (MP, 2 μg/ml) to induce lymphocytes to produce acquired SR, which was manifested by the upregulation of P-gp expression and the decrease in the accumulation of Rh-123 in lymphocytes, and successfully constructed a steroid-resistant splenic lymphocyte lupus mouse (SLC/MP) model [49]. Then, we evaluated the powerful ability of PNS to surmount the SR of SLCs/MP cells as an effective reversal agent. Cell apoptosis analysis implied that the apoptotic cells were significantly reduced in the SLCs/MP cells after PNS treatment, which showed that PNS could obviously improve the MP-induced cytotoxicity to SLCs/MP cells. CD69, a costimulatory signal of T cell proliferation, could more accurately reflect the immunosuppressive effect of steroids. In accordance with the apoptosis experiment, compared with the stimulation group of MP alone, the combination of a high dose of PNS significantly reduced the expression of CD69 in SLCs/MP cells. Moreover, a rhodamine-123 (Rh-123) efflux assay was performed to detect the inhibition of the P-gp efflux pump by PNS and to assess the interaction between PNS and Rh-123. The flow cytometry assay showed that PNS dramatically reversed the accumulation of the P-gp substrate Rh-123 in a concentration-dependent manner. To go a step further, P-gp expression was examined. The expression level of P-gp was markedly suppressed in the SLCs/MP cells treated with 200 μg/ml PNS. In summary, PNS reversed the SR of SLCs/MP cells by reducing the transport capacity and expression of P-gp, thus increasing intracellular steroid accumulation.

SIRT1 has been reported to regulated drug resistance through the deacetylation of its downstream targets, including FoxO1, AKT [50], proliferator-activated receptor gamma coactivator 1-alpha (PGC-1α) [51], CREB [52] and β-catenin [53]. Previous studies have shown that the SIRT1/FoxO1/MDR1 pathway is hyperactivated in many tumours and mediates the multidrug resistance of tumour cells [54]. To verify the existence of this pathway in the SR of SLE, we observed changes in FoxO1 and MDR1 after SIRT1 knockout or plasmid transfection. We found that the concentration of FoxO1 in the nucleus, the expression level of the MDR1 gene and the level of RS changed in accordance with the SIRT1 level in SLCS/MP cells, which indicated that the SIRT1/FoxO1/MDR1 signalling pathway existed in SLCS/MP cells. To further elucidate why PNS inhibited the expression level of P-gp, we used SIRT1 siRNA-transfected SLCs/MP cells to construct a low SIRT1 expression model as a positive control for the downregulation of SIRT1 gene expression, and SLCs/MP cells were used as a negative control. In this study, the effect of PNS on the downregulation of SIRT1 mRNA level was similar to that of SIRT1 siRNA, and when the expression level of SIRT1 mRNA was downregulated, the protein level of SIRT1 was also correspondingly decreased, suggesting that the action site of PNS on the downregulation of SIRT1 level was before translation. After the downregulation of SIRT1 by PNS, the nuclear FoxO1 concentration and *MDR1* mRNA and P-gp levels were simultaneously decreased, suggesting that Panax notosaponins reduced the deacetylation of FoxO1 by changing the SIRT1 level, leading to a reduction in the nuclear Foxo1 concentration, downregulation of *MDR1* gene transcription and downregulation of its expression product P-gp. Thus, PNS reversed the P-gp-mediated SR by suppressing the SIRT1/FoxO1/MDR1 signalling cascade.

An increased frequency of Th17 cells was found in lupus, together with a positive correlation to the SLEDAI [55]. Th17 cells are related to SR. In this study, we first studied the mechanism of PNS reversing SR of lupus from the level of overall spleen lymphocytes, and then further explored the main effector cells of PNS reversing SR. We detected the expression of P-gp in different T-cells phenotypes from SLCs and SLCs/MP and found that the level of P-gp was no difference in CD8 + T cells, Th1, Th2, and Treg cells from SLCs and SLCs/MP except in Th17 cells. Further experimental results show that PNS significantly decreased the expression of P-gp in Th17 cells from SLCs/MP cells compared with untreated SLCs/MP cells. Therefore, Th17 might be the main effector cell of PNS reversing SR in lupus.

## Conclusions

**Fig. 7:**
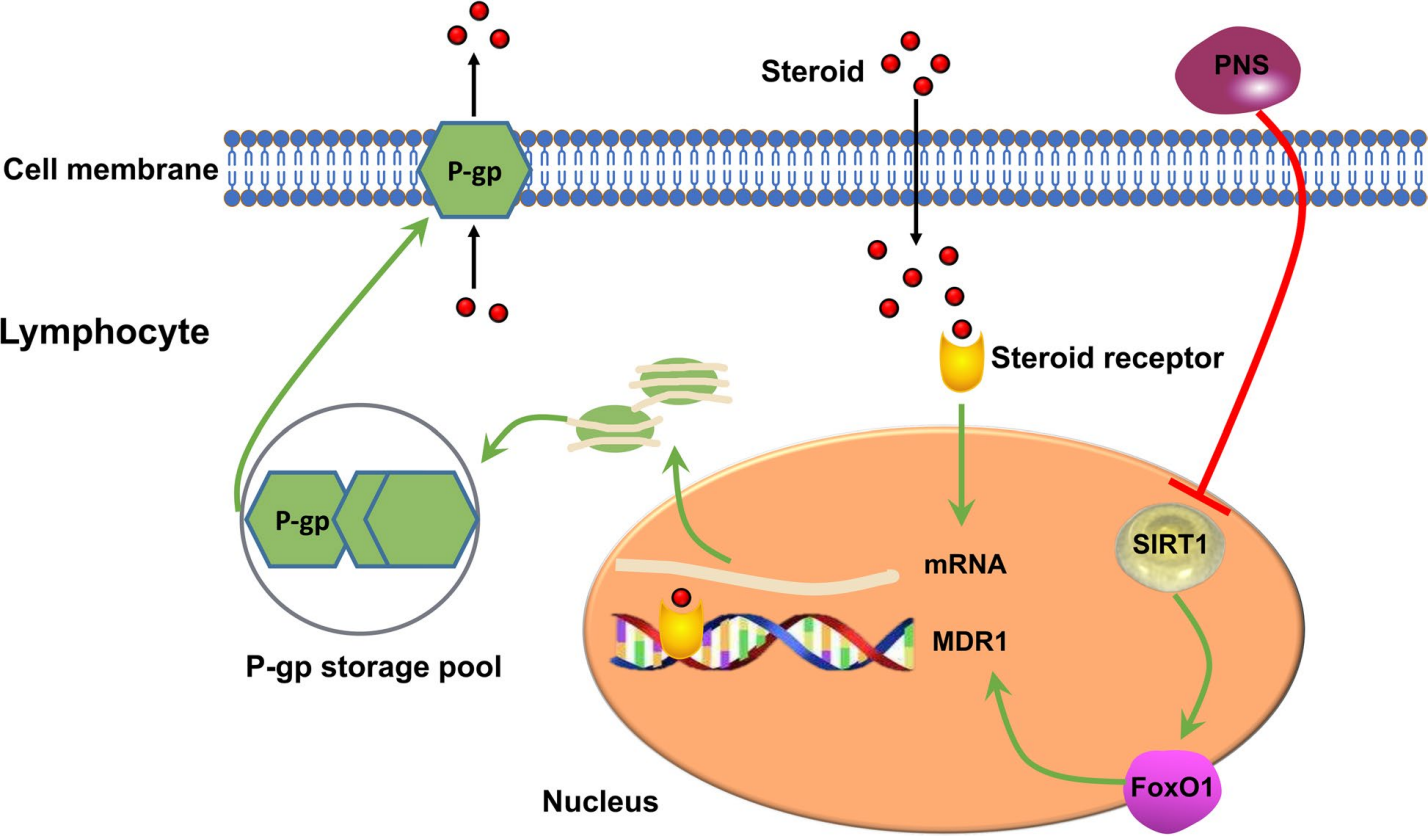
Schematic illustrations of putative signalling mechanisms of PNS in affecting steroid effects (The image in this figure is our own)

Overall, we first revealed that PNS has synergistic and attenuatory effects on steroid therapy in LN. In addition, the potential association of the reversal of P-gp-mediated SR and the SIRT1/FoxO1/MDR1 signalling cascade was identified, which might be the major mechanism involved in the reversal of SR by PNS in SLCs/MP cells (Fig. 7). Finally, We explored Th17 might be the main effector cell of of PNS reversing SR in lupus.

## Supplementary Information

**Additional file 1: Supplementary figure.** The proportion of different T cells phenotypes in SLCs and SLCs/MP cells. (A) The proportion of CD8+ and CD4+ T cells in SLCs and SLCs/MP cells. (B) The proportion of Th1 and Th2 cells in SLCs and SLCs/MP cells. (C) The proportion of Th17 cells in SLCs and SLCs/MP cells. (D) The proportion of Treg cells in SLCs and SLCs/MP cells.

**Additional file 2.**

## Data Availability

The datasets used and analysed during the current study are available from the corresponding author, Ying Lu, on reasonable request.

## Abbreviations

SLE: Systemic lupus erythematosus
LN: Lupus nephritis
SR: Steroid resistance
P-gp: P-glycoprotein
GR: Glucocorticoid receptor
MIF: Macrophage migration inhibitory factor
MKP-1: MAPK phosphatase-1
MDR1: Multidrug resistance 1
SIRT1: Silent information regulation factor related enzyme 1
FoxO1: Forkhead box-containing protein of the O subfamily 1
PNS: Panax notoginseng saponin
Rh-123: Rhodamine-123
MP: Methylprednisolone
SRLM: Steroid resistance lupus mouse
SLCs: Splenic lymphocytes of lupus mouse
SLCs/MP: Steroid resistance splenic lymphocytes of lupus mouse
PGC-1α: Proliferator-activated receptor gamma coactivator 1-alpha

## References

[1] Abdwani R, Al Shaqsi L, Al-Zakwani I (2018). Neonatal and Obstetrical Outcomes of Pregnancies in Systemic Lupus Erythematosus. Oman Med J.

[2] Tipton CM, Hom JR, Fucile CF, Rosenberg AF, Sanz I (2018). Understanding B-cell activation and autoantibody repertoire selection in systemic lupus erythematosus: A B-cell immunomics approach. Immunol Rev.

[3] Ruiz-Irastorza G, Danza A, Khamashta M (2012). Glucocorticoid use and abuse in SLE. Rheumatology (Oxford).

[4] Strehl C, Ehlers L, Gaber T, Buttgereit F (2019). Glucocorticoids—All-Rounders Tackling the Versatile Players of the Immune System. Front Immunol.

[5] Gao H, Wang Q, Yu X, Liu J, Bai S, Feng J, Wu B (2018). Molecular mechanisms of glucocorticoid resistance in systemic lupus erythematosus: A review Life Sci..

[6] Rodriguez JM, Monsalves-Alvarez M, Henriquez S, Llanos MN, Troncoso R (2016). Glucocorticoid resistance in chronic diseases. Steroids.

[7] Kansal A, Tripathi D, Rai MK, Agarwal V (2016). Persistent expression and function of P-glycoprotein on peripheral blood lymphocytes identifies corticosteroid resistance in patients with systemic lupus erythematosus. Clin Rheumatol.

[8] Wang FF, Zhu LA, Zou YQ, Zheng H, Yang CD, Shen N, Chen SL, Lu LJ (2012). New insights into the role and mechanism of macrophage migration inhibitory factor in steroid-resistant patients with systemic lupus erythematosus. Arthritis Res Ther.

[9] Van Winsen LM, Hooper-van Veen T, van Rossum EF (2007). Glucocorticoid receptor gene polymorphisms associated with more aggressive diseasephenotype in MS. J Neuroimmunol.

[10] Roger T, Chanson AL, Knaup-Reymond M, Calandra T (2005). Macrophage migration inhibitory factor promotes innate immune responses by suppressing glucocorticoid- induced expression of mitogen-activated protein kinase phosphatase-1. Eur J Immunol.

[11] Schewitz-Bowers LP, Lait PJ, Copland DA (2015). Glucocorticoid-resistant Th17 cells are selectively attenuated by cyclosporine. A Proc Natl Acad Sci.

[12] Cosmi L, De Palma R, Santarlasci V (2008). Human interleukin 17-producing cells originate from a CD161+CD4+ T cell precursor. J Exp Med.

[13] Chowdhary VR (2020). When doing the right thing is wrong–drug efflux pumps in steroid-resistant nephrotic syndrome. Indian J Rheumatol.

[14] Singh H, Prasad N, Misra DP, Jaiswal AK, Agarwal V (2020). P-glycoprotein and/or Histone Deacetylase 2 Regulates Steroid Responsiveness in Childhood Nephrotic Syndrome. Indian J Rheumatol.

[15] Singh H, Agarwal V, Chaturvedi S, Misra DP, Jaiswal AK, Prasad N (2019). Reciprocal Relationship Between HDAC2 and P-Glycoprotein/MRP-1 and Their Role in Steroid Resistance in Childhood Nephrotic Syndrome. Front Pharmacol.

[16] Yuan Z, Shi X, Qiu Y, Jia T, Yuan X, Zou Y, Liu C, Yuan Y, Xu K, Yin P (2017). Reversal of P-gp-mediated multidrug resistance in colon cancer by cinobufagin. Oncol Rep.

[17] Ma X, Hu M, Wang H, Li J (2018). Discovery of traditional Chinese medicine monomers and their synthetic intermediates. Eur J Med Chem.

[18] Badr HS, El-Hawy MA, Helwa MA (2016). P-Glycoprotein Activity in Steroid-Responsive vs. Steroid-Resistant Nephrotic Syndrome Indian J Pediatr.

[19] Perez-Guerrero EE, Gamez-Nava JI, Muñoz-Valle JF (2018). Serum levels of P-glycoprotein and persistence of disease activity despite treatment in patients with systemic lupus erythematosus. Clin Exp Med.

[20] Choi HK, Cho KB, Phuong NT (2013). SIRT1-mediated FoxO1 deacetylation is essential for multidrug resistance-associated protein 2 expression in tamoxifen-resistant breast cancer cells. Mol Pharm.

[21] Kang H, Oka S, Lee DY (2017). Sirt1 carboxyl-domain is an ATP-repressible domain that is transferrable to other proteins. Nat Commun.

[22] Wang Y, Zhang L, Che X, Li W, Liu Z, Jiang J (2018). Roles of SIRT1/FoxO1/SREBP-1 in the development of progestin resistance in endometrial cancer. Arch Gynecol Obstet.

[23] Yan X, Yu A, Zheng H, Wang S, He Y, Wang L (2019). Calycosin-7-O-beta-D-glucoside Attenuates OGD/R-Induced Damage by Preventing Oxidative Stress and Neuronal Apoptosis via the SIRT1/FOXO1/PGC-1alpha Pathway in HT22 Cells. Neural Plast.

[24] Oh WK, Cho KB, Hien TT (2010). Amurensin G, a Potent Natural SIRT1 Inhibitor, Rescues Doxorubicin Responsiveness via Down-Regulation of Multi-drug Resistance 1. Mol Pharmacol.

[25] Ashktorab H, Soleimani A, Singh G (2019). Saffron: The Golden Spice with Therapeutic Properties on Digestive Diseases. Nutrients.

[26] Boozari M, Hosseinzadeh H (2021). Natural products for COVID-19 prevention and treatment regarding to previous coronavirus infections and novel studies. Phytother Res.

[27] Murali C, Mudgil P, Gan CY (2021). Camel whey protein hydrolysates induced G2/M cellcycle arrest in human colorectal carcinoma. Sci Rep.

[28] Huang W, Li X, Wang D (2020). Curcumin reduces LPS-induced septic acute kidney injury through suppression of lncRNA PVT1 in mice. Life Sci..

[29] Yang Q, Wang P, Cui J, Wang W, Chen Y, Zhang T (2016). Panax notoginseng saponins attenuate lung cancer growth in part through modulating the level of Met/miR-222 axis. JEthnopharmacol.

[30] Meng L, Lin J, Huang Q (2019). Panax notoginseng Saponins Attenuate Oxygen-Glucose Deprivation/Reoxygenation-Induced Injury in Human SH-SY5Y Cells by Regulating the Expression of Inflammatory Factors through miR-155. Biol Pharm Bull.

[31] Zhang M, Guan Y, Xu J, Qin J, Li C, Ma X, Zhang Z, Zhang B, Tang J (2019). Evaluating the protective mechanism of panax notoginseng saponins against oxidative stress damage by quantifying the biomechanical properties of single cell. AnalChim Acta.

[32] Guo X, Sun W, Luo G (2019). Panax notoginseng saponins alleviate skeletal muscle insulin resistance by regulating the IRS1-PI3K-AKT signaling pathway and GLUT4 expression. FEBS Open Bio.

[33] Chian S, Zhao Y, Xu M (2019). Ginsenoside Rd reverses cisplatin resistance in non-small-cell lung cancer A549 cells by downregulating the nuclear factor erythroid 2-related factor 2 pathway. Anticancer Drugs.

[34] Li W, Li G, She W, Hu X, Wu X (2019). Targeted antitumor activity of Ginsenoside (Rg1) in paclitaxel-resistant human nasopharyngeal cancer cells are mediated through activation of autophagic cell death, cell apoptosis, endogenous ROS production, S phase cell cycle arrest and inhibition of m-TOR/PI3K/AKT signalling pathway. J BUON.

[35] Zhang H, Gong J, Zhang H, Kong D (2015). Induction of apoptosis and reversal of permeability glycoprotein-mediated multidrug resistance of MCF-7/ADM by ginsenoside Rh2. Int J Clin Exp Pathol.

[36] Li C, Sun BQ, Gai XD (2014). Compounds from Chinese herbal medicines as reversal agents for P-glycoprotein-mediated multidrug resistance in tumours. Clin Transl Oncol.

[37] Feng SL, Luo HB, Cai L (2020). Ginsenoside Rg5 overcomes chemotherapeutic multidrug resistance mediated by ABCB1 transporter: in vitro and In vivo study. J Ginseng Res.

[38] Li J, Liu D, Wu J (2016). Ginsenoside Rg1 attenuates ultraviolet B-induced glucocortisides resistance in keratinocytes via Nrf2/HDAC2 signalling. Sci Rep.

[39] Lu Y, Yang R, Zhang H, Zhu X (2011). The effects of Panax Notoginseng Saponins on P-glycoprotein and the function of glucocorticoid in lymphocytes of systemic lupus erythematosus patients. Chin J Of Rheumatology.

[40] Ding W, Xu Z, Wu R, Tong Y, Lu Y (2018). PNS Regulate SIRT1 on Steroid Resistance and Lipid Metabolism in Spleen Lymphocyte of LN Mice by Acting on PPAR Gamma. Journal of Zhejiang Chinese Medical University.

[41] Tsujimura S, Saito K, Nakayamada S, Nakano K, Tanaka Y (2005). Clinical relevance of the expression of P-glycoprotein on peripheral blood lymphocytes to steroid resistance in patients with systemic lupus erythematosus. Arthritis Rheum.

[42] Lu Z, Li J, Ji J, Gu Z, Da Z (2019). Altered peripheral lymphocyte subsets in untreated systemic lupus erythematosus patients with infections. Braz J Med Bio Res..

[43] Tsujimura S, Saito K, Tokunaga M (2005). Overcoming treatment unresponsiveness mediated by P-glycoprotein overexpression on lymphocytes in refractory active systemic lupus erythematosus. Mod Rheumatol.

[44] Joshi P, Vishwakarma RA, Bharate SB (2017). Natural alkaloids as P-gp inhibitors for multidrug resistance reversal in cancer. Eur J Med Chem.

[45] Lopes-Rodrigues V, Sousa E, Vasconcelos MH (2016). Curcumin as a Modulator of P-Glycoprotein in Cancer: Challenges and Perspectives. Pharmaceuticals (Basel).

[46] Liu C, Gong Q, Chen T, Lv J, Feng Z, Liu P, Deng Z (2018). Treatment with 20(S)-ginsenoside Rg3 reverses multidrug resistance in A549/DDP xenograft tumors. Oncol Lett.

[47] Zhou X, Wang A, Wang L, Yin J, Wang L, Di L, Shan L, Wu X, Wang Y (2019). A Danshensu-Tetramethylpyrazine Conjugate DT-010 Overcomes Multidrug Resistance in Human Breast Cancer. Front Pharmacol.

[48] Wang H, Jia XH, Chen JR, Wang JY, Li YJ (2016). Osthole shows the potential to overcome P-glycoproteinmediated multidrug resistance in human myelogenous leuke-mia K562/ADM cells by inhibiting the PI3K/Akt signaling pathway. Oncol Rep..

[49] Yang R, Lu Y, Yang J, Zhu X, Zhang Y, Lin Y (2010). Study on the relationship between glucocorticoid resistance and P-glycoprotein in lymphocytes induced by low dose methylprednisolone. Chin J Clin Pharmacol.

[50] Ling S, Li J, Shan Q, Dai H (2017). USP22 mediates the multidrug resistance of hepatocellular carcinoma via the SIRT1/AKT/MRP1 signaling pathway. Mol Oncol.

[51] Xu R, Luo X, Ye X (2021). SIRT1/PGC-1α/PPAR-γ Correlate With Hypoxia-Induced Chemoresistance in Non-Small Cell Lung Cancer. Front Oncol..

[52] Zhang L, Guo X, Zhang D (2017). Upregulated miR-132 in Lgr5+ gastric cancer stem cell-like cells contributes to cisplatin-resistance via SIRT1/CREB/ABCG2 signaling pathway. Mol Carcinog.

[53] Jin X, Wei Y, Liu Y (2019). Resveratrol promotes sensitization to Doxorubicin by inhibiting epithelial-mesenchymal transition and modulating SIRT1/β-catenin signaling pathway in breast cancer. Cancer Med.

[54] Ceballos MP, Decándido G, Quiroga AD (2018). Inhibition of sirtuins 1 and 2 impairs cell survival and migration and modulates the expression of P-glycoprotein and MRP3 in hepatocellular carcinoma cell lines. Toxicol Lett.

[55] Liu MF, Wang CR (2014). Increased Th17 cells in flow cytometer-sorted CD45RO-positive memory CD4 T cells from patients with systemic lupus erythematosus. Lupus Sci. Med..

