# Supplementary material for: Panax notoginseng saponins reverse P-gp-mediated steroid resistance in lupus: involvement in the suppression of the SIRT1/FoxO1/MDR1 signalling pathway in lymphocytes
Source: BMC Complement Med Ther. 2022 Jan 12;22:13. doi: 10.1186/s12906-021-03499-5 (PMC8756704; doi:10.1186/s12906-021-03499-5)
Supplement: Supplementary file 1 — Additional file 1: Supplementary figure. The proportion of different T cells phenotypes in SLCs and SLCs/MP cells. (A) The proportion of CD8+ and CD4+ T cells in SLCs and SLCs/MP cells. (B) The proportion of Th1 and Th2 cells in SLCs and SLCs/MP cells. (C) The proportion of Th17 cells in SLCs and SLCs/MP cells. (D) The proportion of Treg cells in SLCs and SLCs/MP cells. [file 12906_2021_3499_MOESM1_ESM.doc]

**Supplementary figures**

**
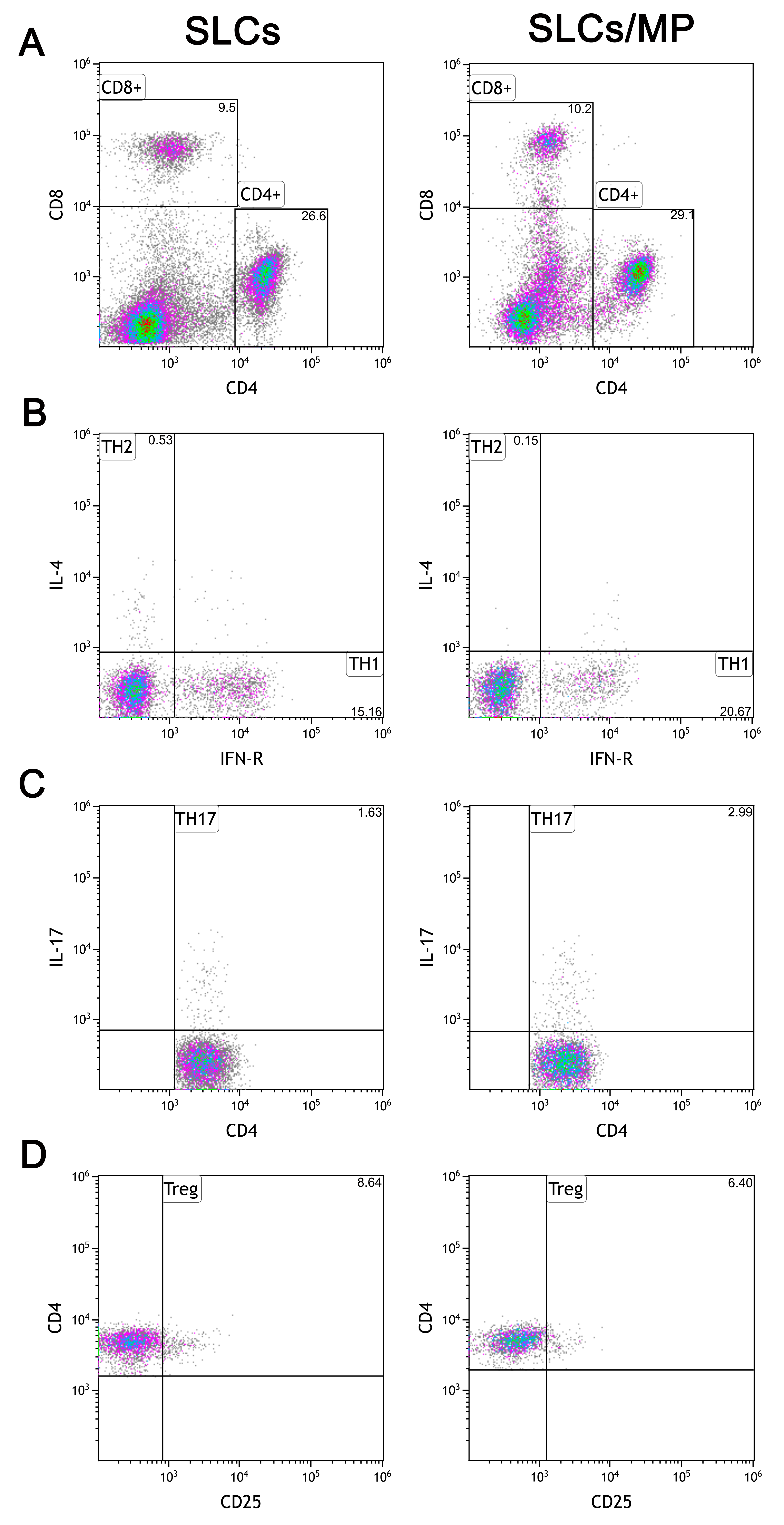
**

**Supplementary figure.** The proportion of different T cells phenotypes in SLCs and SLCs/MP cells. (A) The proportion of CD8+ and CD4+ T cells in SLCs and SLCs/MP cells. (B) The proportion of Th1 and Th2 cells in SLCs and SLCs/MP cells. (C) The proportion of Th17 cells in SLCs and SLCs/MP cells. (D) The proportion of Treg cells in SLCs and SLCs/MP cells.
