# Supplementary figures and images for: Panax notoginseng saponins reverse P-gp-mediated steroid resistance in lupus: involvement in the suppression of the SIRT1/FoxO1/MDR1 signalling pathway in lymphocytes
Source: BMC Complement Med Ther. 2022 Jan 12;22:13. doi: 10.1186/s12906-021-03499-5 (PMC8756704; doi:10.1186/s12906-021-03499-5)

**SIRT1(Figure 4A)**

**
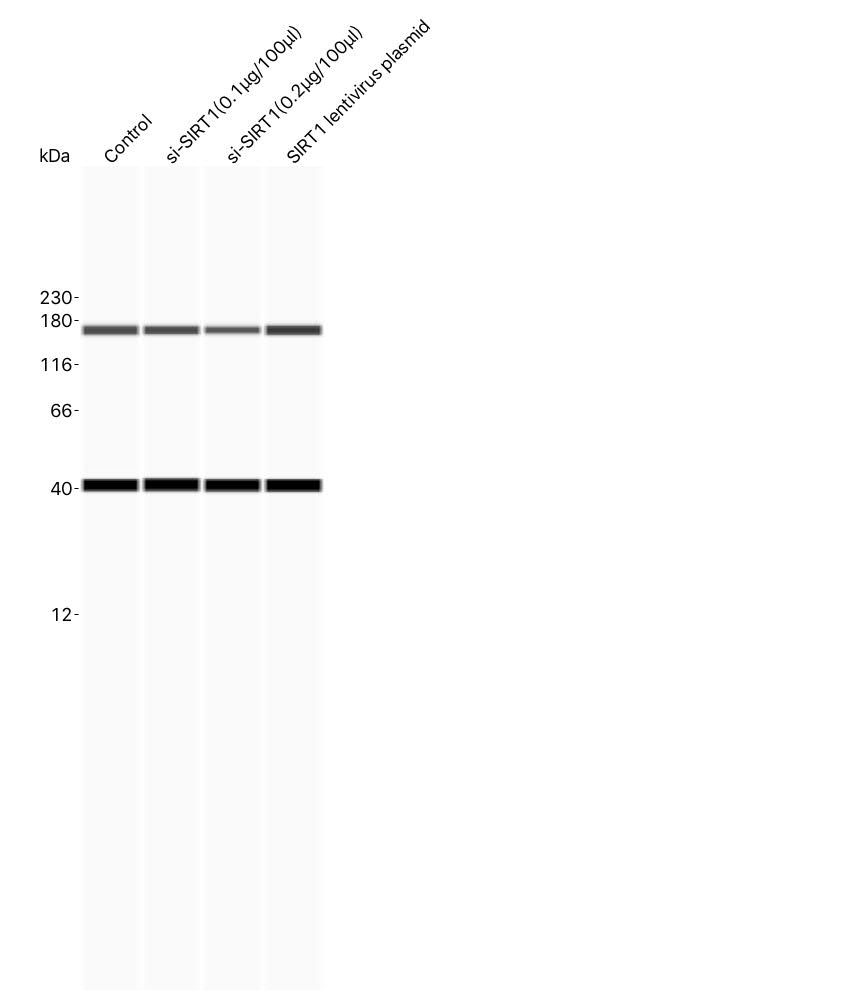
**

**FoxO1(Figure 4A)**


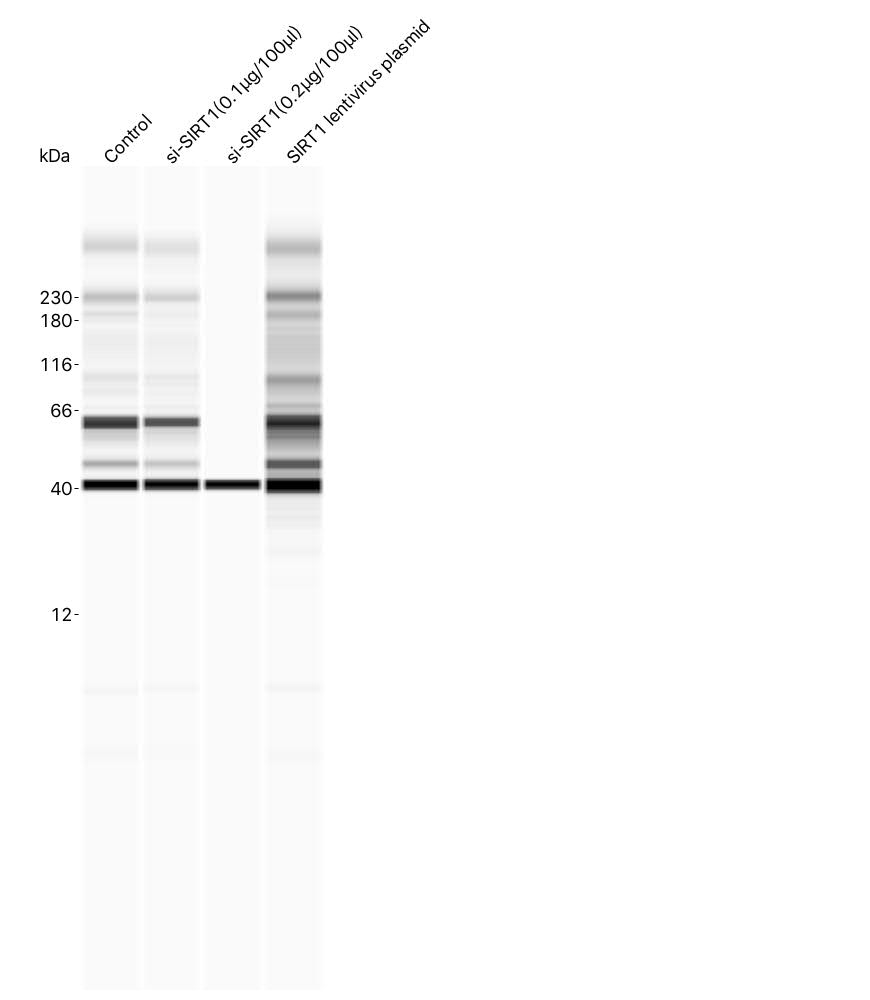


**SIRT1(Figure 5A)**


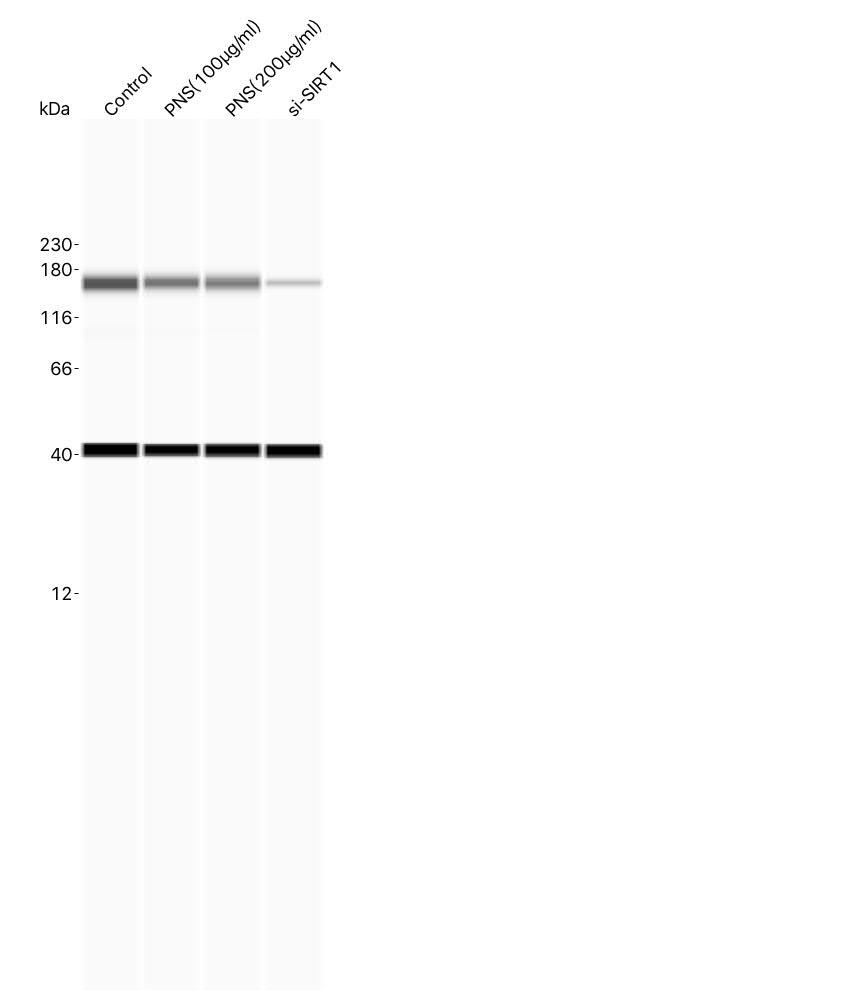


**FoxO1(Figure 5A)**


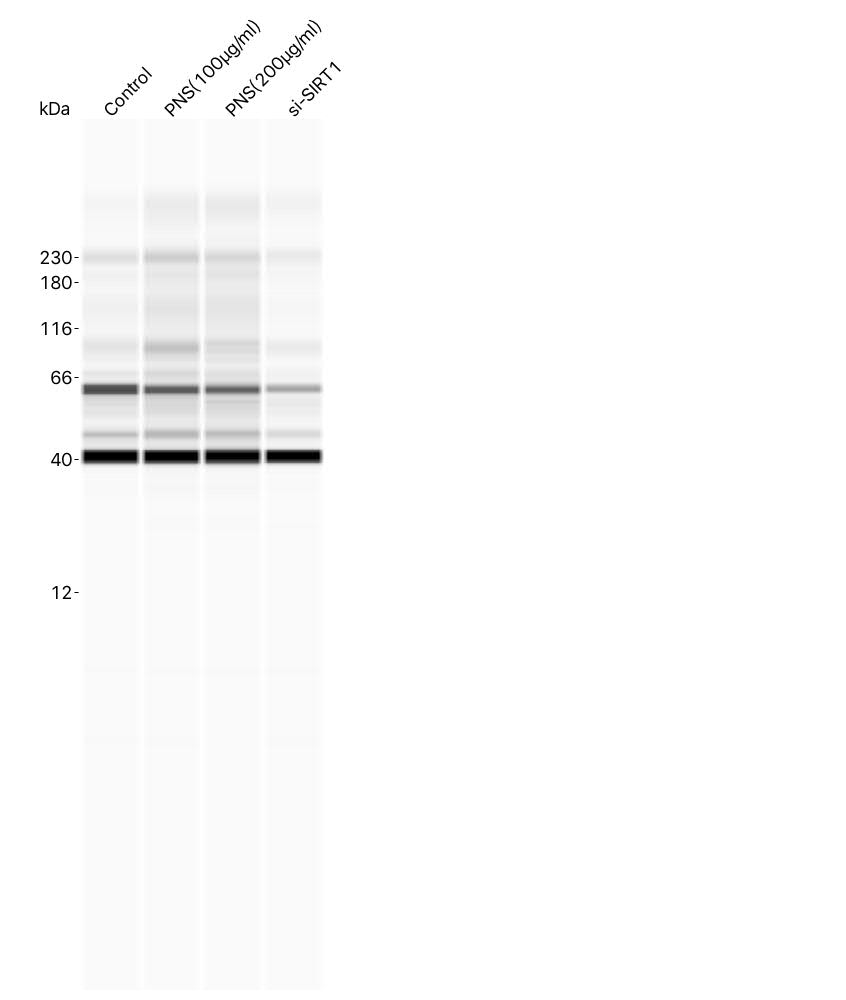

Supplement: Supplementary file 2 — Additional file 2. [file 12906_2021_3499_MOESM2_ESM.doc]
